# Shotgun Metagenomics Identifies in a Cross‐Sectional Setting Improved Plaque Microbiome Biomarkers for Peri‐Implant Diseases

**DOI:** 10.1111/jcpe.14121

**Published:** 2025-06-04

**Authors:** Paolo Ghensi, Vitor Heidrich, Davide Bazzani, Francesco Asnicar, Federica Armanini, Alberto Bertelle, Federico Dell'Acqua, Ester Dellasega, Romina Waldner, Daniela Vicentini, Mattia Bolzan, Lorenzo Trevisiol, Cristiano Tomasi, Edoardo Pasolli, Nicola Segata

**Affiliations:** ^1^ PreBiomics S.r.l. Trento Italy; ^2^ Department CIBIO University of Trento Trento Italy; ^3^ Private Practice Trentino‐Alto Adige Italy; ^4^ CISMed – Centre for Medical Sciences Trento Italy; ^5^ Department of Periodontology, Institute of Odontology, Sahlgrenska Academy University of Gothenburg Gothenburg Sweden; ^6^ Department of Agricultural Sciences University of Naples Federico II Portici Italy

**Keywords:** microbiome, mucositis, peri‐implantitis, peri‐implant diseases, shotgun metagenomics

## Abstract

**Aim:**

This observational study aimed to verify and improve the predictive value of plaque microbiome of patients with dental implant for peri‐implant diseases.

**Materials and Methods:**

Patients were included in one of the following study groups according to the health status of their dental implants: (a) healthy, (b) affected by mucositis and (c) affected by peri‐implantitis. From each patient, submucosal plaque microbiome samples were collected from the considered dental implant and from a contralateral healthy implant/tooth. After shotgun metagenomic sequencing, the plaque microbiome was profiled taxonomically and functionally with MetaPhlAn 4 and HUMAnN 3, respectively. Taxonomic and functional profiles were fed into machine‐learning models, which were then evaluated with cross‐validation to assess the extent to which the plaque microbiome could be used to pinpoint peri‐implant diseases.

**Results:**

Shotgun metagenomics sequencing was performed for a total of 158 samples spanning 102 individuals. Four‐hundred and forty‐seven prokaryotic species were identified as part of the peri‐implant microbiome, 34% of which were currently uncharacterized species. At the community level, the peri‐implant microbiome differed according to the health status of the implant (*p* ≤ 0.006 for all pairwise comparisons) but this was site‐specific, as healthy contralateral sites showed no discriminating microbiome features. Peri‐implantitis microbiomes further showed lower inter‐subject variability than healthy plaque microbiomes (*p* < 0.001), while mucositis‐associated microbiomes were in the middle of the continuum between health and peri‐implantitis. Each health condition was associated with a strong signature of taxonomic and functional microbiome biomarkers (log_10_ LDA score ≥ 2.5), 30% and 13% of which represented uncharacterized microbial functions and unknown species, respectively. Distinct *Fusobacterium nucleatum* clades were associated with implant status, highlighting the subspecies *F. nucleatum*′s functional and phenotypic diversity. Machine‐learning models trained on taxonomic or functional plaque microbiome profiles were highly accurate in differentiating clinical groups (AUC = 0.78–0.96) and highlighted the extent to which the peri‐implant microbiome is associated with peri‐implant clinical parameters (AUC = 0.79–0.87).

**Conclusions:**

Plaque microbiome profiling with shotgun metagenomics revealed consistent associations between microbiome composition and peri‐implant diseases. In addition to pointing to peri‐implant‐associated microbes, warranting further mechanistic studies, we showed high‐resolution plaque microbiome evaluation via metagenomics as an effective tool. Its utility within protocols for clinical management of peri‐implant diseases should be explored in the future.

## 1 Introduction

Rehabilitation of edentulous and partially edentulous patients often includes the use of dental implants. At the same time, it is widely established that peri‐implant diseases are a constantly growing concern, as they represent a challenging biomedical and economic problem (Heitz‐Mayfield 2008; Lindhe et al. 2008; Zitzmann and Berglundh 2008; Junior, Romito, and Shibli 2019). They are defined as plaque‐associated pathological conditions occurring in tissues surrounding dental implants and include two different entities: peri‐implant mucositis and peri‐implantitis (Ramanauskaite, Becker, and Schwarz 2018; Renvert et al. 2018). Peri‐implant mucositis is a reversible inflammation of the peri‐implant tissues limited to the mucosa, while peri‐implantitis is characterized by mucosal inflammation signs and supporting bone loss (Caton et al. 2018). The literature agrees in estimating that about half of implant patients have at least one implant affected by mucositis and that one in every five has at least one implant affected by peri‐implantitis (Derks and Tomasi 2015; Derks et al. 2016; Berglundh et al. 2018; Schwarz, Derks, and Monje 2018; Heitz‐Mayfield and Salvi 2018).

The 2017 World Workshop on the Classification of Periodontal and Peri‐Implant Diseases and Conditions has identified microbial plaque as the main aetiological factor of mucositis and peri‐implantitis. At the same time, it has underlined how, to date, no specific bacteria associated with peri‐implant diseases have been defined (Berglundh et al. 2018). It is now evident that in order to develop and improve diagnostic, preventive and therapeutic approaches, a thorough profiling and a more comprehensive understanding of the oral plaque microbiome associated with peri‐implant diseases is a crucial and necessary step.

Most of the studies available to date to characterize the role of the plaque microbiome in peri‐implant diseases have relied on 16S rRNA gene sequencing (Carvalho et al. 2023; de Melo et al. 2020), which, however, is unable to identify most microbes at the species level and cannot profile the overall functional potential of the microbial community (Quince et al. 2017; Tyson et al. 2004; Venter et al. 2004). To overcome these limitations and enable strain‐level profiling, in our previous work we applied, for the first time, shotgun metagenomics to sequence and deeply analyse the plaque microbiome in different peri‐implant health and disease conditions in a large sample of implant patients (Ghensi et al. 2020). We were able to find strong microbial signatures for mucositis and peri‐implantitis and defined the so‐called peri‐implantitis‐related complex (PiRC). Our data suggested the peri‐implantitis microbiome seems to be strictly site‐specific, while mucositis is enriched for *Fusobacterium nucleatum* acting as a keystone colonizer, with strain‐level profiling identifying a previously uncharacterized subspecies of *F. nucleatum* as particularly associated with disease.

We hypothesized that the known associations between peri‐implant microbiome and peri‐implant conditions could be further confirmed and substantially expanded by leveraging the unprecedented level of detail provided by recent advances in shotgun metagenomic data analysis, supporting the use of the peri‐implant microbiome as a potential diagnosis‐supporting tool in this setting. In this study, we have (a) expanded the sample size of implant patients from our previous study, (b) considered only implants in the three clinical conditions (healthy vs. mucositis vs. peri‐implantitis), (c) applied state‐of‐the‐art computational tools capable of profiling metagenomes at unprecedented resolution, including identification of unknown genes (HUMAnN 3) and species (MetaPhlAn 4) (Beghini et al. 2021; Blanco‐Míguez et al. 2023), (d) associated peri‐implant plaque microbiome with clinical parameters to estimate how strongly they are linked and (e) applied artificial intelligence techniques on metagenomic profiles for evaluating the potential of the approach as part of future applications in the clinical setting.

## 2 Materials and Methods

### 2.1 Study Design, Ethical Approval and Patient Recruitment

This study was designed as an observational study with a protocol approved by the Ethics Committee of the University of Trento (no. 2015‐024). The study was conducted between June 2016 and February 2018 in accordance with the guidelines of the World Medical Association Declaration of Helsinki. Subjects recruited in this study were selected from a population of patients who had completed dental implant therapy and received regular maintenance visits (on average at least one check‐up per year including professional oral hygiene procedure) after prosthetic rehabilitation in six different private practices in the province of Trentino (Italy). Potential participants were informed about the aims, potential risks and benefits of this study, and were assured that their participation was voluntary. All participants recruited signed a written informed consent prior to their inclusion, and the extracted data in the present study was anonymized.

The inclusion criteria were (i) good medical health as evidenced by the medical history, (ii) being at least 18 years of age, (iii) having at least 8 teeth, (iv) having at least one functioning oral implant restored with crowns or prostheses for at least 1 year and (v) manifested willingness to participate in the study. Exclusion criteria included (i) current pregnancy or lactation, (ii) infection with human immunodeficiency virus, (iii) current use of immunosuppressant medications, bisphosphonates or steroids (iv) use of chlorhexidine mouthwash or gel during the previous 2 weeks, (v) oral prophylactic procedures within the preceding 3 months and (vi) intake of systemic antibiotics or probiotics within the past 6 months.

### 2.2 Study Groups

Patients consenting to be included in the study were included in one of the following study groups according to the state of health of their dental implants: (a) healthy (H, patients with at least one healthy implant and no implants with mucositis or peri‐implantitis); (b) mucositis (M, patients with at least one implant with mucositis and no implants with peri‐implantitis); and (c) peri‐implantitis (P, patients with at least one implant with peri‐implantitis). The selection and inclusion of patients in one of the groups was based on radiographic evaluation of the marginal bone level, clinical signs of inflammation and/or presence of suppuration (SUP) according to the criteria delineated by the Consensus Report on Peri‐implant Diseases available at that time (Lindhe et al. 2008). In detail, peri‐implant was considered to be in a healthy state when the implant was surrounded by healthy soft tissue as determined by the absence of bleeding on probing (BOP) or SUP and visible/detectable radiographic bone loss. Implants with only clinical signs of inflammation in one or more sites (redness, swelling, bleeding, SUP) and absence of radiographic bone loss following functional loading were classified as peri‐implant mucositis, whereas implants with the presence in one or more sites of both clinical inflammation and radiographic evidence of > 2 mm bone loss since the prosthesis installation (i.e., at least 1 year after loading) were diagnosed as peri‐implantitis.

### 2.3 Data Collection and Clinical Examination

Six experienced dentists examined all patients. Before commencing the study, meetings were organized in order to instruct all dentists on a common protocol for the examination, collection and measurement procedures. The demographic parameters of sex and age and a comprehensive medical and dental history were recorded for each enrolled volunteer, followed by a full‐mouth periodontal and implant clinical examination and, if necessary, a site‐specific radiographic examination. Medical history comprised information about smoking habit, diabetes, autoimmune diseases or other systemic diseases, alcohol consumption and medications taken. Dental history comprised information about current and past periodontal status, number of remaining teeth, number of implants, previous peri‐implantitis, frequency of home oral care, hours since the last toothbrushing and chlorhexidine usage. Clinical parameters included whether the sampled site was an implant or a tooth (for the contralateral site), site of sampling, diagnosis of implant age (time from installation), implant system used and nature of reconstruction (single implant, fixed or removable), type of implant retention (screw, cement, conometric), radiographic peri‐implant bone loss, width of the keratinized mucosa, as well as periodontal probing depth (PPD), plaque index (PI), BOP and SUP. The latter four parameters were measured in each patient at the buccal, mesial, lingual and distal sites of the experimental implant (healthy/mucositis/peri‐implantitis) and of a healthy contralateral implant (if present) or a healthy tooth. PI, BOP and SUP were recorded on a binary scale (presence/absence) for each surface and PPD was measured to the nearest millimetre on the scale. Intra‐oral peri‐apical radiographs were obtained with the parallel technique, and peri‐implant bone levels were recorded by measuring the distance from the implant shoulder to the first visible bone to implant contact at the mesial and distal aspect of each implant. In the case of mucositis and peri‐implantitis, any eventual subsequent therapy was noted. All patients were pseudo‐anonymized in the clinic by assigning a unique patient ID to all subjects. All downstream analyses were performed using anonymous IDs and anonymized metadata. The mapping between patients and IDs was stored and kept uniquely at the clinic, and only the person responsible at center was allowed to have access to it.

### 2.4 Sample Collection

Plaque samples were collected by a limited number of trained dentists (one for each study center) in order to minimize potential biases in the sampling methodology across the different examiners. Follow‐up meetings were organized every 3 months to ensure consistency of the sampling, and statistical tests on the results were applied to verify the absence of strong dentist‐specific batch effects. Microbiome samples were collected with a non‐invasive procedure by adopting a sampling protocol previously described (Ghensi et al. 2020). Samples were collected with replicates (two per site) from a single implant and from the healthy contralateral implant/tooth (if present, a healthy implant was preferred as contralateral healthy site) for each patient in each study group. If multiple implants with the same tested condition were present in a patient, one a single implant was randomly selected for sampling. The sampling sites were isolated using cotton rolls to prevent contamination with saliva and gently dried with an air syringe, and supra‐mucosal and supra‐gingival plaque was removed using sterile cotton pellets. Submucosal and subgingival plaque samples were taken from the deepest probing site at each selected implant and tooth with individual sterile titanium Gracey curettes. Replicates were sampled only after the interruption of any eventual bleeding in order to avoid contamination of the microbiological sample. Curettes were preferred to sterile paper points to avoid potential contaminating bacterial DNA associated with paper points (van der Horst et al. 2013). After the collection, samples were immediately placed in separate 1.5‐mL Eppendorf microcentrifuge tubes (Eppendorf, Hamburg, Germany) containing sterile SCF‐1 buffer solution (50 mM Tris–HCl, pH 7.5; 1 mM EDTA, pH 8.0; 0.5% Tween‐20) (Tett et al. 2017) and frozen at −80 °C until further analysis.

### 2.5 DNA Extraction and Illumina Shotgun Sequencing

Total genomic DNA was isolated using the Qiagen Power Soil Pro Kit (Qiagen, Hilden, Germany), following the manufacturer's protocol (except for the addition of an enzymatic disruption step for complete lysis of gram‐positive and gram‐negative bacteria). Isolated DNA was stored at −20°C. Laboratory control extractions were also performed on prepared sample buffer to assess any potential contaminants. Each extracted sample was first quantified, and when there was sufficient DNA material (> 1 ng), libraries were prepared using the Illumina DNA prep Kit (Illumina Inc., San Diego, CA, USA) following the manufacturer's protocol. Technical replicates were used only for the cases in which the first sampling did not yield enough DNA. Libraries were sequenced (2 × 150 bp reads) on the Illumina NovaSeq‐6000 platform. Eleven patients (eight in the healthy group, one in the mucositis group and two in the peri‐implantitis group) were excluded because of failure in DNA extraction or sequencing library preparation. Overall, 158 libraries underwent shotgun metagenomic sequencing.

### 2.6 Sequence Preprocessing, Quality Control and Taxonomic and Functional Potential Profiling

Sequenced samples were subject to preprocessing as implemented in https://github.com/SegataLab/preprocessing to perform quality control. The protocol consisted of a read‐level quality control step, a screening of contaminant host sequences step and a split and sorting of cleaned reads step. In the initial quality control, low‐quality reads (quality score < Q20), fragmented short reads (< 75 bp) and reads with more than five ambiguous nucleotides were removed. Human DNA (hg19 human genome release) and Illumina spike‐ins were then removed using BowTie2 (Langmead and Salzberg 2012). Out of the 158 metagenomes generated, 20 samples showing insufficient non‐human reads depth (< 10 Mbases) or high levels of potential skin contamination (*Cutibacterium acnes* relative abundance > 10%) were discarded, 11 of which were implant samples (characteristics of the excluded implants are summarized in Table S1). We used MetaPhlAn version 4 (Blanco‐Míguez et al. 2023) with default parameter settings for taxonomic characterization of the sampled microbial community at the resolution of single species‐level genome bins (SGBs) and including uncharacterized species with no sequenced representative. HUMAnN version 3 (Beghini et al. 2021) was used to generate normalized pathway and gene family relative abundance profiles according to the metagenome gene contents.

### 2.7 Statistical Analysis

Considering that it is impossible to know beforehand the number of microbiome features that will be revealed by the metagenomic profiling methods employed, we could not perform a priori power analysis for this study. It is in any case reassuring that we are here expanding the sample size of a prior powered study (Ghensi et al. 2020). We performed biomarker discovery using LEfSe (Segata et al. 2011) on MetaPhlAn 4 taxonomic relative abundance profiles and HUMAnN 3 gene family relative abundance profiles. Alpha‐diversity, beta‐diversity and multidimensional scaling (MDS) ordination were performed via custom python scripts based on Scipy (version 1.7.3) and Scikit‐learn (version 1.0.2) (Pasolli et al. 2016; Varoquaux et al. 2015). PERMANOVA was performed using the Scikit‐bio python library (version 0.5.6) with 1000 permutations. We used the FDR correction adopting the Benjamini–Hochberg approach implemented in the python library Statsmodel (version 0.13.1). Beta‐diversity was computed using the Bray–Curtis dissimilarity index on raw relative abundances. Two‐tailed Wilcoxon rank‐sum test was used for comparisons unless otherwise stated.

### 2.8 Machine‐Learning Analysis

The machine‐learning framework applied to this work is based upon a Scikit‐Learn (version 1.0.1) implementation of random forest (Breiman 2001). This method was chosen because it achieved the highest consistency and lowest overfitting on metagenomic datasets as reported in several studies including those for the analysis of the link between the gut microbiome and colorectal cancer (Thomas et al. 2019). We applied this framework to MetaPhlAn 4 SGB‐level relative abundances and HUMAnN 3 gene family relative abundances. We set the number of estimators of the random forest classifier to 1000, while we chose a maximum number of features to consider when looking for the best split of 30% of the feature set for MetaPhlAn 4 and of the square root of the size of the feature set for HUMAnN 3. We set the maximum number of samples in each leaf to 5 while using the default settings of Scikit‐Learn (version 1.0.1) for the other hyperparameters. We trained and tested the model in 20 times repeated 10‐fold stratified cross‐validations (Pasolli et al. 2016; Asnicar et al. 2024). In brief, this approach will divide the data into 10 random folds, and for each iteration will use 9 of them as training data and the remaining as testing data. This is then repeated 20 times so that multiple random folds can be formed and evaluated. To assess the performance of the classifier, for each of 200 classification tasks (20 runs × 10 folds), the receiver operating characteristic (ROC) curve is computed. We finally evaluate the predictions through the averaged area under the ROC curve (AUC) value.

## 3 Results

### 3.1 An Expanded Metagenomic Dataset of the Peri‐Implant Microbiome

The study was conceived and designed as a cross‐sectional study to investigate the link between the plaque microbiome and peri‐implant health. According to the protocol, a submucosal plaque sample was collected from each patient from an implant in one of the following three conditions: healthy conditions (H), affected by mucositis (M) or affected by peri‐implantitis (P). In each patient from whom we sampled a test implant, we also sampled a healthy contralateral site (a healthy implant if present, a healthy tooth otherwise) as an intra‐subject control.

In the present study, we considered a total of 102 patients (H: 40, M: 29 or P: 33; 51 males, 51 females; mean age 62.54 ± 9.85 years), 72 of whom have been analysed in our previous study (Ghensi et al. 2020), in which earlier versions of computational analysis pipelines that could not account for uncharacterized species were adopted. Overall, quality‐controlled (see Section 2) shotgun metagenomics data were produced for a total of 138 samples (856 Gbases in total), spanning 87/102 patients.

This cohort was, on average, 62.28 ± 9.84 years old and included 46 males and 41 females, 16 of whom were smokers (Table 1). The analysed population was composed of 31 patients with a healthy implant (H), 25 patients with mucositis (M) and 31 patients with peri‐implantitis (P). No significant differences between the three groups were found for age (ANOVA, *p* = 0.89), sex (Pearson chi‐square, *p* = 0.31), history of severe periodontitis (Pearson chi‐square, *p* = 0.06), smoking habit (Pearson chi‐square, *p* = 0.09), diabetes (Pearson chi‐square, *p* = 0.30), number of functioning implants (*p* = 0.60), number of residual teeth (*p* = 0.16), previous peri‐implantitis (Pearson chi‐square, *p* = 0.13) or frequency of home oral care (*p* = 0.07).

**TABLE 1:** Demographic and clinical characteristics of the cohort (*n* = 87).

	Healthy	Mucositis	Peri‐implantitis	*p*
No. of subjects	31 (36%)	25 (29%)	31 (36%)	—
Age, mean (SD)	61.6 (9.1)	62.5 (11.3)	62.8 (9.8)	0.89
Sex (M/F)	18/13 (21/14%)	10/15 (11/17%)	18/13 (21/14%)	0.31
History of severe periodontitis (Y/N)	8/23 (9/26%)	13/12 (15/14%)	17/14 (20/16%)	0.06
Smoking (Y/N)	2/29 (2/33%)	7/18 (8/21%)	7/24 (8/28%)	0.09
Diabetes cases	2 (2%)	0 (0%)	3 (3%)	0.30
No. of implants, mean (SD)	3.7 (3.0)	4.0 (2.2)	4.4 (3.1)	0.60
No. of teeth, mean (SD)	20.8 (7.1)	18.3 (8.3)	16.9 (8.4)	0.16
Previous peri‐implantitis (Y/N)	2/26 (2/30%)	3/18 (3/21%)	8/22 (9/25%)	0.13
Frequency of home oral care, mean (SD)	2.1 (0.9)	2.4 (0.9)	2.0 (0.7)	0.07

*Note:* For categorical variables, percentages in relation to the entire cohort are indicated. Statistical tests used to compare clinical groups are indicated in the main text.

For eight patients, only contralateral samples were retained, and therefore 79 out of 87 analysed patients contributed with test implant samples to the analysed dataset (26 H, 24 M and 29 P). Extensive clinical data were registered both for test implants (Table S2) and contralateral implants or teeth (Table S3). The 79 test implants were in function for on average 8.9 ± 5.3 years (range: 1–24 years), 38 of which (48.1%) were located in the mandible, and belonged to nine different implant systems (Table S4). The mean PPD at the test sampled sites was 4.7 ± 2.2 mm, with 34 out of 79 (43.0%) presenting PPD ≥ 5 mm and 54 (68%) showing signs of bleeding.

When comparing clinical parameters for the test implants between implant groups, a significant difference was observed for PPD (ANOVA, *p* < 0.001), BOP (Pearson chi‐square, *p* < 0.001), SUP (Pearson chi‐square, *p* < 0.001) and bone loss (ANOVA, *p* < 0.001), with all these parameters significantly higher in P implants compared to H implants. PI was, however, non‐significantly different (Pearson chi‐square, *p* = 0.58) among groups.

### 3.2 Microbial Profiles of Healthy and Diseased Peri‐Implant Sites

**FIGURE 1:**
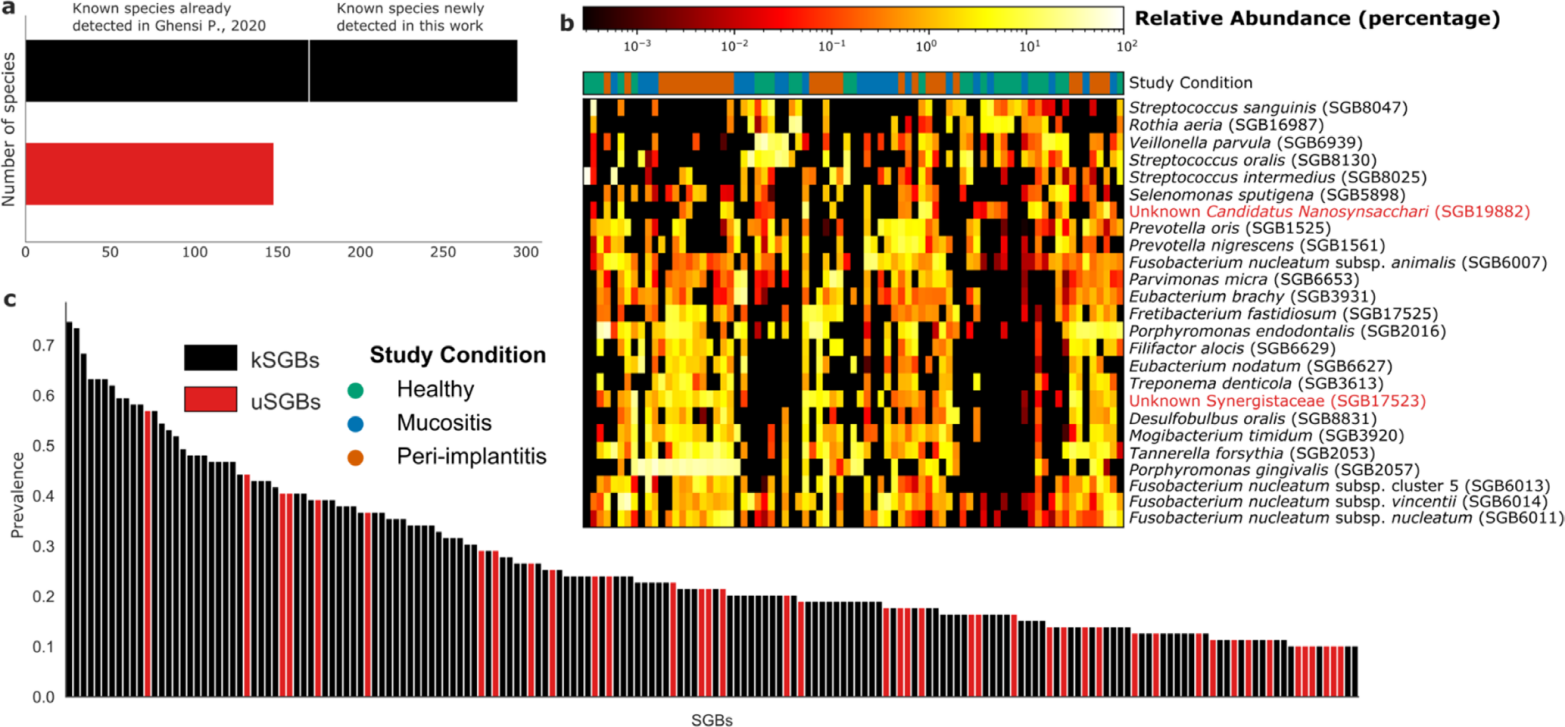
Overview of the taxonomic composition of the peri‐implant microbiome. (a) Comparison between the amount of unknown species‐level genome bins (uSGBs, red) and known species‐level genome bins (kSGBs, black); kSGBs are further divided between those already detected in Ghensi et al. (2020) and those detected for the first time in this work. (b) Abundance profiles across samples of the 25 most abundant SGBs (according to their 25th percentile stratified by study condition). uSGBs are reported in red and represent new unknown species. (c) Ordered prevalence of the SGBs with > 10% prevalence, divided into kSGBs and uSGBs.

According to the latest version of the profiling tool MetaPhlAn (version 4, see Section 2), the taxonomic composition of the peri‐implant microbiome in the whole population was characterized by a total of 447 species‐level genome bins (in short SGBs, representing single bacterial species) present in at least one sample. Of these 447 SGBs, 297 are known SGBs (kSGBs) and 150 unknown SGBs (uSGBs), representing species yet to be formally named and whose diversity needs to be explored. Among the 297 kSGBs, only 171 were also previously identified by Ghensi et al. (2020) (Figure 1a). Among the 150 uSGBs detected, several are abundant members of the peri‐implant microbiome (55 uSGBs present at ≥ 1% relative abundance in at least two samples), including two uSGBs among the top 25 most abundant SGBs in the peri‐implant microbiome (Figure 1b,c).

### 3.3 A Stronger Microbiome Signature for Peri‐Implantitis Sites Versus Healthy Sites

**FIGURE 2:**
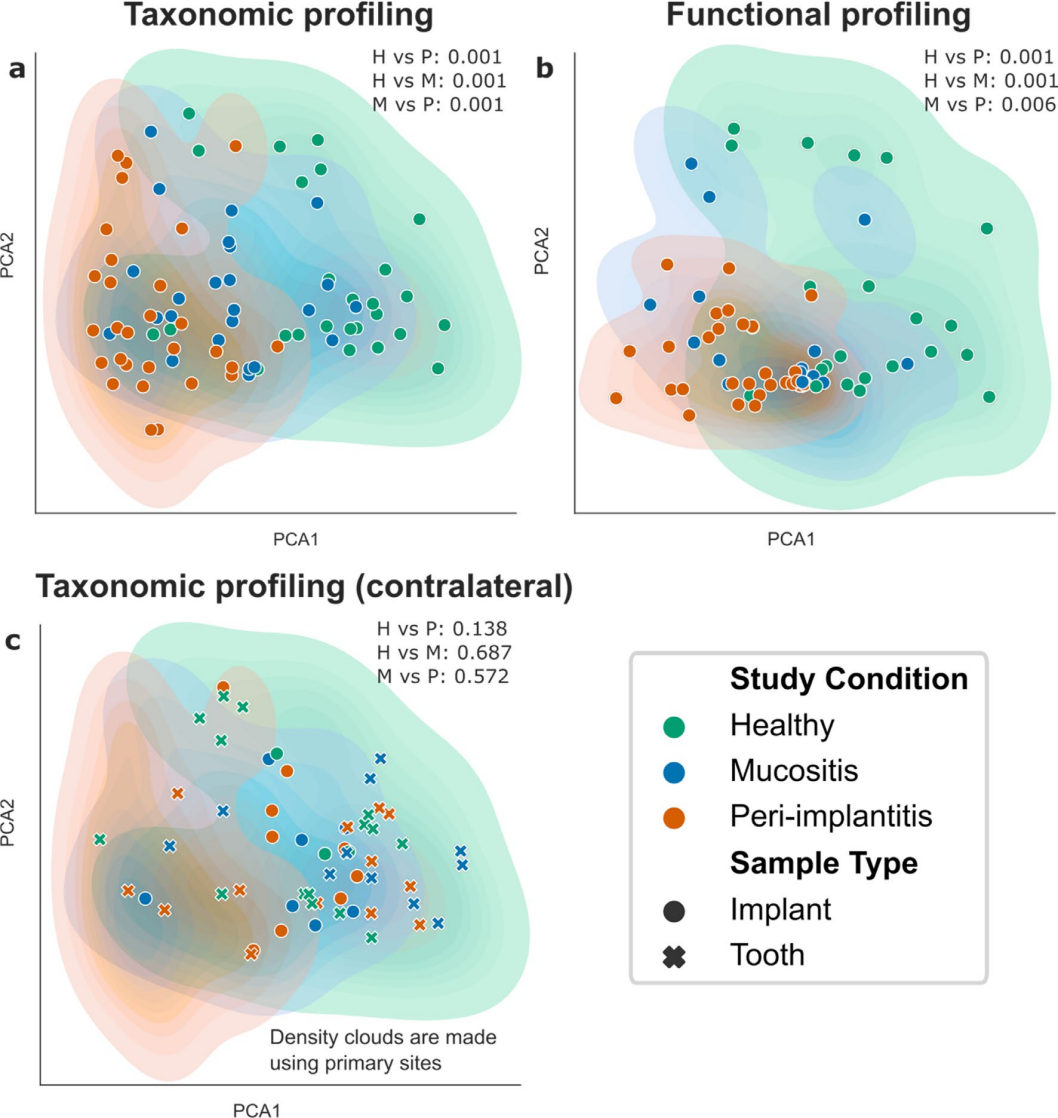
Taxonomic and functional clustering of peri‐implant microbiome samples according to disease state. (a) Multidimensional scaling (MDS) ordination plot of healthy, mucositis and peri‐implantitis sites based on the Bray–Curtis beta‐diversity between microbiome samples. *p*‐values were obtained by PERMANOVA. (b) and (c) report the same type of ordination plots but based on functional abundance profiles (UniProt90 gene family relative abundances) and on the taxonomic profiles of the contralateral samples from implants/teeth.

Quantitative analysis of the plaque microbiome based on both taxonomic compositions via MetaPhlAn 4 (see Section 2) (Figure 2a) and functional profiles via HUMAnN 3 (see Section 2) (Figure 2b) highlighted a distinction between groups both taxonomically and functionally in test sites (PERMANOVA, *p* ≤ 0.006 for all pairwise comparisons). In contrast, samples from contralateral healthy sites clustered together with those from healthy test implants (Figure 2c), with no significant difference between groups among contralateral implants/teeth both taxonomically (PERMANOVA, *p* > 0.13 for all pairwise comparisons) (Figure 2c) and functionally (PERMANOVA, *p* > 0.33 for all pairwise comparisons) (Figure S1).

Despite mucositis samples having distinct microbiome compositions compared to healthy implant and peri‐implantitis samples, a discrete sample clustering distinctive for mucositis was not observed, with mucositis samples showing a level of dispersion comparable to that of healthy implants taxonomically (PERMDISP, *p* = 0.14 for M vs. H) and occupying a microbiome compositional space overlapping with many healthy implant and peri‐implantitis samples (Figure 2a,b). In contrast, H and P sample compositions rarely overlapped (Figure 2a,b), but with P samples showing more well‐defined taxonomic compositions than H implants (PERMDISP, *p* = 0.02 for P vs. H). P samples showing significantly lower compositional variability than other clinical conditions is also clear from both taxonomic (*p* < 0.001 for both P vs. M and P vs. H) and functional (*p* < 0.001 for both P vs. M and P vs. H) beta‐diversity boxplots (Figure S1). Together, these results suggest that fully diseased dental implants converge towards a specific ‘peri‐implantitis microbiome’.

### 3.4 Improved and Novel Microbial Taxonomic Biomarkers of Peri‐Implant Diseases

**FIGURE 3:**
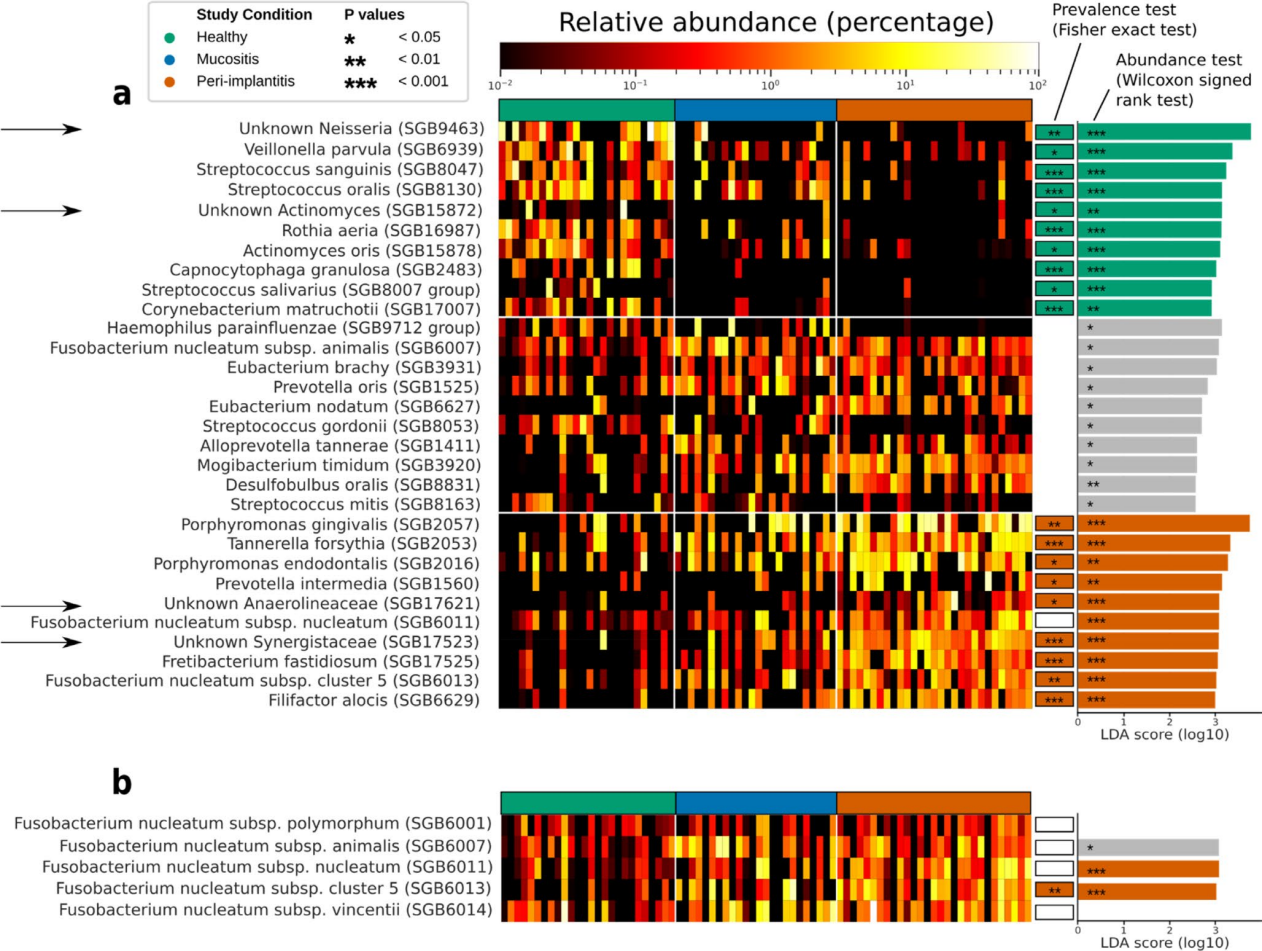
Plaque microbiome biomarkers across disease states. The heatmap (a) reports the relative abundances (log scale) and the effect sizes (the LDA score from LEfSe) of the top 10 species‐level genome bins (SGBs) with the highest effect sizes that characterize each study condition: Health SGBs (health vs. peri‐implantitis, green effect size bars); mucositis SGBs (mucositis vs. health or peri‐implantitis, grey effect size bars): and peri‐implantitis SGBs (peri‐implantitis vs. health, red effect size bars). The *p*‐values of the abundance test performed by LEfSe are shown inside the bar. The rectangular boxes represent the results of the prevalence test (Fisher exact test) between healthy and peri‐implantitis biomarkers: The colour of the box corresponds to the study condition in which the associated species is more prevalent, while the *p*‐values are reported as stars (white boxes are used to indicate species with a non‐significant difference in prevalence between healthy and peri‐implantitis samples). Arrows point to unknown SGBs. Panel (b) specifically highlights the abundance patterns and associations of the five subspecies of *F. nucleatum* .

Corroborating a peri‐implantitis microbiome signature, several SGBs were strongly associated with peri‐implantitis (based on presence and abundance) compared to the other study conditions (Figure 3a). The SGBs found here to be enriched in peri‐implantitis considerably overlap with the set of bacteria recently associated with peri‐implantitis (Ghensi et al. 2020) and the ones traditionally linked to periodontitis (Socransky et al. 1998). These include *Porphyromonas gingivalis* (SGB2057), *Tannerella forsythia* (SGB2053), *Porphyromonas endodontalis* (SGB2016), *Prevotella intermedia* (SGB1560), *Fretibacterium fastidiosum* (SGB17525), as well as two different *Fusobacterium nucleatum* clades: subsp. *nucleatum* (SGB6011) and subsp. cluster 5 (SGB6013). Previously unreported peri‐implantitis‐associated species were also found, though, including two uSGBs. These two uSGBs belong to the families Anaerolineaceae and Synergistaceae and are ranked fifth and seventh, respectively, in terms of effect size among the SGBs enriched in peri‐implantitis in comparison to healthy implants (Figure 3a). This is similar to the observed in healthy implants, in which an uSGB belonging to the *Neisseria* genus and a uSGB belonging to the *Actinomyces* genus were among the most discriminative SGBs (first and fifth, respectively) for healthy implant microbiome.

In contrast with the observed peri‐implantitis microbial signature, the list of SGBs enriched in mucositis samples shows only one SGB previously reported to have any association with periodontitis and/or peri‐implantitis, namely *F. nucleatum* subsp. *animalis* (SGB6007) (Figure 3a), which is one of the five *F. nucleatum* SGBs observed in our cohort. Interestingly, two of those *F. nucleatum* SGBs (SGB6011 and SGB6013, but not SGB6007) are, instead, highly discriminative for peri‐implantitis (Figure 3a). In fact, *F. nucleatum* subsp. cluster 5 (SGB6013) was not only overall highly abundant among peri‐implantitis samples (mean relative abundance 1.96%) but also highly (prevalence 83%) and significantly more prevalent (Fisher exact test, *p* < 0.01 for P vs. H) in peri‐implantitis. Conversely, other two *F. nucleatum* subspecies, namely *F. nucleatum* subsp. *polymorphum* (SGB6001) and *F. nucleatum* subsp. *vincentii* (SGB6014), were consistently present and abundant in samples from all three clinical conditions (Figure 3b). *F. nucleatum* is thus a diverse species including several clades (captured by the definition of SGBs) with likely distinct roles along the mucositis–peri‐implantitis axis.

Beyond taxonomic compositions, microbiome functions were also clearly linked to each condition, again with no overlap between gene families associated with mucositis and peri‐implantitis (Figure S2). Overall, most of the gene families enriched in healthy or diseased states are related to transposase activity and are largely uncharacterized.

### 3.5 A Machine‐Learning Classifier Can Discriminate Between Peri‐Implant Disease States Based on Microbiome Profiles

**FIGURE 4:**
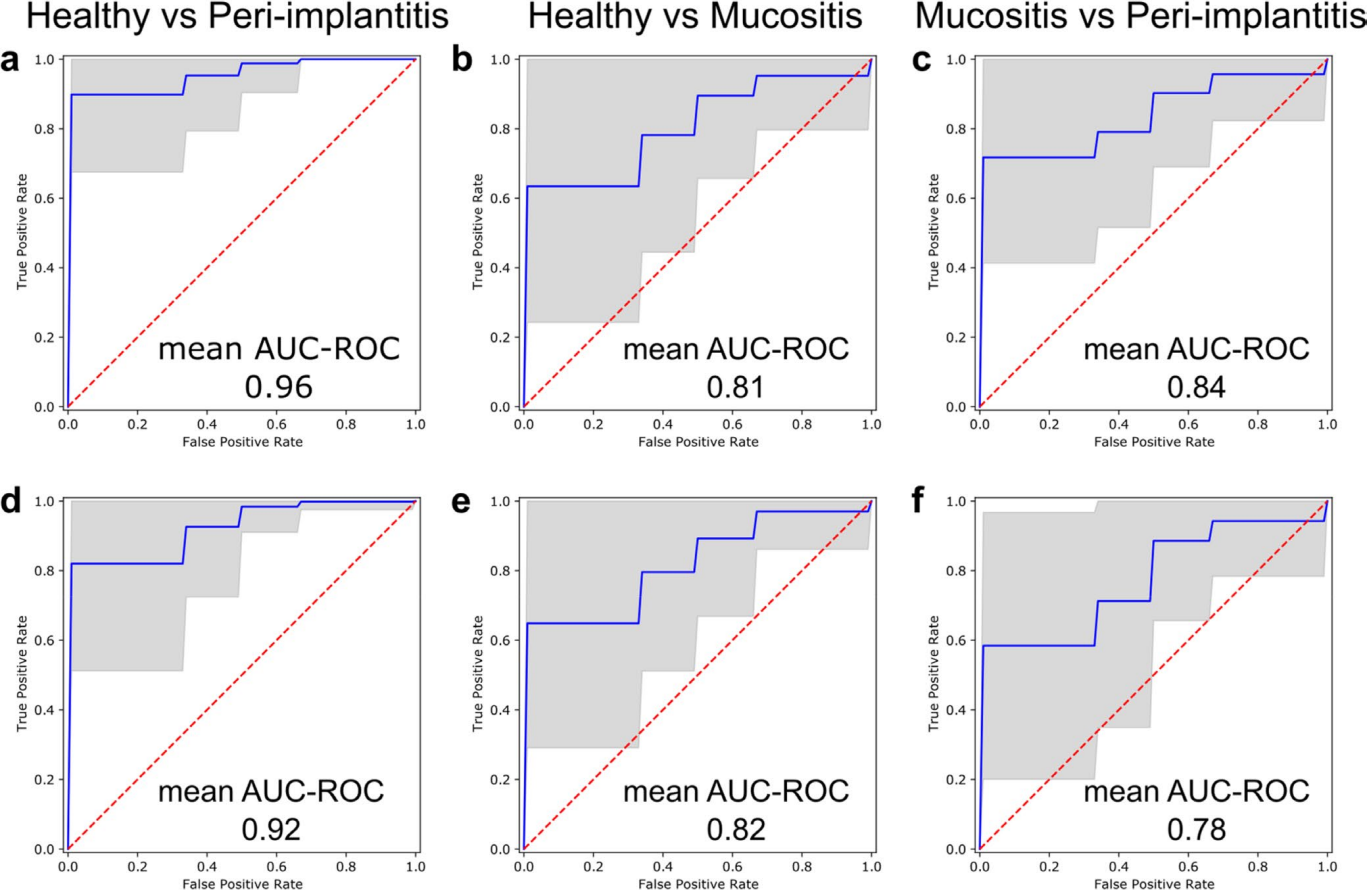
Microbiome‐based machine‐learning classification of disease states. Evaluation of the performance of the random forest classifier in classifying samples into the study conditions based on taxonomic features (SGB relative abundances, a–c) and functional features (UniRef90 relative abundances, d–f). ROC curves and AUC values are averaged across testing folds. Grey areas represent the interval of ±1 standard deviation away from the mean.

We then assessed the potential of the peri‐implant microbiome for discriminating between the three study conditions. Using a validated machine‐learning approach based on the random forest classifier (Pasolli et al. 2016; Asnicar et al. 2024) and a cross‐validation setting (see Section 2), we found that peri‐implantitis can be predicted with very high accuracy from SGB‐level taxonomic compositions (AUC = 0.96) (Figure 4a). Mucositis also possessed a discriminant microbiome but with considerably less precision and recall for the task of predicting mucositis versus health (AUC = 0.81) (Figure 4b). Discriminating P and M samples could also be done accurately (AUC = 0.84) and slightly better than M versus H, highlighting the strong specificity of the peri‐implantitis microbiome even in the face of another diseased state (Figure 4c). Even though they were not significantly different between groups in our study (possibly due to limited sample size), we also evaluated the potential effect of confounders (age, sex, smoking, diabetes, history of severe periodontitis, history of peri‐implantitis, frequency of home oral care, number of implants) on these machine‐learning classifications. We found that such variables alone perform poorly in the task of classifying implant clinical conditions and that they do not improve microbiome‐based models (Figure S3), indicating a likely minor effect of confounders in the microbiome associations we report.

When considering microbiome functional profiles (gene families) for training the classifiers, we achieved accuracies that were comparable with those obtained for taxonomic profiles (Figure 4d–f). This indicates that the SGBs with discriminative power for telling apart the three study conditions using taxonomic compositions are likely associated with the gene families useful to predict conditions based on functional profiles.

### 3.6 Composition of the Plaque Microbiome Is Associated With Peri‐Implant Clinical Parameters

**FIGURE 5:**
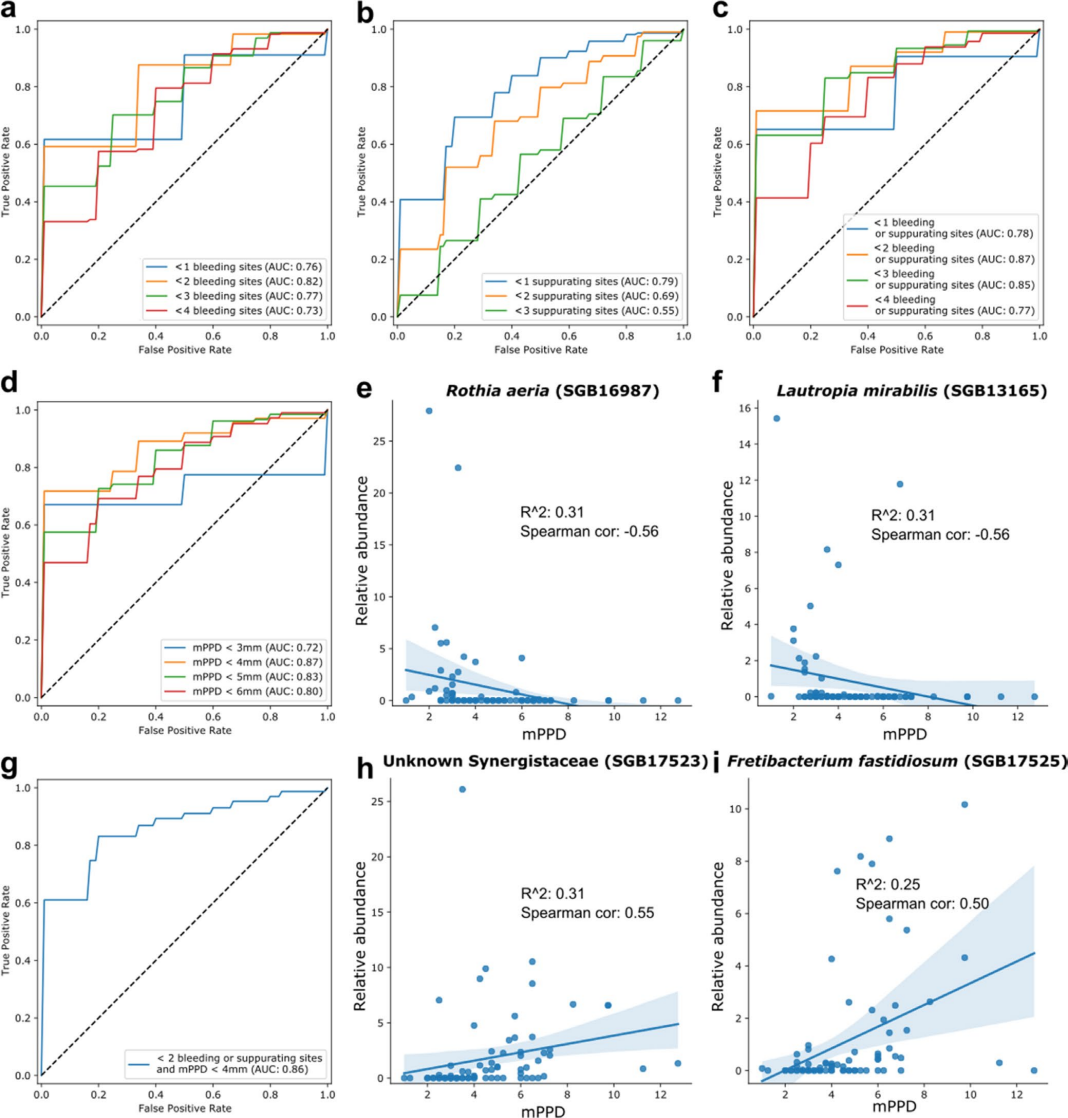
Association between plaque microbiome and peri‐implant clinical parameters using machine learning. Receiver operating characteristic (ROC) curves and mean area under the ROC curve (AUC) values for the cross‐validation performance of the random forest classifier based on SGB relative abundances for predictingars shown: Different thresholds of bleeding on probing (BOP) positive sites (a), different thresholds of suppurating positive sites (b), different thresholds of bop and/or SUP sites (c), different thresholds of mPPD (d) and combined clinical signs of health (g). The scatterplots show the significant correlations between SGB relative abundances and mPPD (e, f, h, i); Spearman correlation coefficients and *R* ^2^ values are reported.

After showing that the plaque microbiome is strongly associated with dental implant disease states, we assessed whether it was also linked to peri‐implant clinical parameters (BOP, SUP and PPD) that are routinely used in the clinical practice to monitor dental implant condition (Figure 5). We found that plaque taxonomic composition is associated with BOP (AUC = 0.73–0.82, depending on the number of sites threshold) and SUP (AUC = 0.55–0.79, depending on the number of sites threshold). If considered separately, the presence of < 2 BOP sites (Figure 5a) or < 1 SUP site (Figure 5b) can be discriminated with the highest accuracy (AUC = 0.82 and AUC = 0.79, respectively). If BOP and SUP are considered together (Figure 5c), the highest accuracy is achieved for discriminating implants with < 2 BOP and/or SUP sites (AUC = 0.87) (Figure 5c).

Mean PPD around multiple sites (mPPD) can similarly be predicted from plaque microbiome (AUC = 0.72–0.87, depending on the depth threshold) (Figure 5d), with taxonomic compositions being better able to discriminate mPPD at the threshold of < 4 mm (AUC = 0.87), but also showing high accuracy at the threshold of < 5 mm (AUC = 0.83). The high overall accuracy of using taxonomic compositions to predict mPPD is linked to the strong correlations found between mPPD and some SGB relative abundances (Figure 5e,f,h,i). In detail, *Rothia aeria* (SGB16987) and *Lautropia mirabilis* (SGB13165) are negatively correlated with mPPD (Spearman, −0.56 for both), while uSGBs belonging to the Synergistaceae family (SGB17523) and *Fretibacterium fastidiosum* (SGB17525) are positively correlated with mPPD (Spearman, 0.55 and 0.50, respectively).

Finally, we considered BOP, SUP and mPPD together (Figure 5g). The clinical condition best discriminable by the plaque microbiome taxonomic composition has an mPPD < 4 mm and < 2 BOP and/or SUP sites (AUC = 0.86). Evidence from machine‐learning models that the plaque microbiome associates with either separate or combined implant health parameters reinforces the strong extent to which the peri‐implant microbiome composition is linked to the peri‐implant environment.

## 4 Discussion

In this study, we described strong and specific associations between the oral plaque microbiome and peri‐implant diseases. This was achieved by (i) expanding a previously analysed cohort of dental implant patients and (ii) combining shotgun metagenomics with advanced computational techniques, which together allow microbiome profiling at unprecedented levels of detail. The greater statistical power and higher microbiome resolution of our study enabled us to further explore the potential of the plaque microbiome as a diagnostic tool to assist in the maintenance of dental implant health.

In our previous work (Ghensi et al. 2020), we profiled the peri‐implant microbiome functionally and taxonomically using HUMAnN 2 (Franzosa et al. 2018) and MetaPhlAn 2 (Truong et al. 2015), respectively. Although they represented the state of the art at that time, significant improvements in HUMAnN 3 (Beghini et al. 2021) and MetaPhlAn 4 (Blanco‐Míguez et al. 2023) justified re‐analysis of the data, expanded by new plaque samples, with these recently developed tools. The current version of HUMAnN benefits from more recent databases and algorithmic enhancements, which make it significantly more accurate than its predecessor (Beghini et al. 2021). The current version of MetaPhlAn leverages de novo metagenomic assembly to also allow the quantification of species not present in isolate genome databases (i.e., unknown species) (Blanco‐Míguez et al. 2023). As more than a third of peri‐implant microbes represented yet‐to‐be‐characteriezd species (uSGBs), MetaPhlAn 4 was key to unravelling the full breadth of peri‐implant microbiome diversity. Of note, some uSGBs were among the most important biomarkers of dental implant health and disease, suggestive of a role of yet‐to‐be‐characterized species in mucositis and peri‐implantitis. Two uSGBs, belonging to the families Anaerolineaceae and Synergistaceae and representing species yet to be formally named and whose diversity needs to be explored, are in fact ranked fifth and seventh between the bacterial species most discriminative for peri‐implantitis.

Another advantage of the SGB approach adopted by MetaPhlAn 4 is that profiling at the species level is performed in a genomically consistent manner and it is not directly affected by taxonomic inconsistencies. We previously reported that *F. nucleatum* seemed to be a key species in the plaque ecological transition from mucositis to peri‐implantitis because it was a biomarker of both conditions. Here, however, by leveraging the SGB approach implemented in MetaPhlAn 4, we found that different *F. nucleatum* SGBs (which consistently matched previously defined *F. nucleatum* subspecies) were associated either with mucositis ( *F. nucleatum* subsp. *animalis*) or peri‐implantitis ( *F. nucleatum* subsp. *nucleatum* and *F. nucleatum* subsp. cluster 5), while other *F. nucleatum* SGBs were equally prevalent both in health and diseased states. This highlights that it is crucial to employ a metagenomic taxonomic profiling tool able to discriminate between *F. nucleatum* subclades, as they have diverging roles in the development and progression of peri‐implant diseases.

Despite the very limited congruence between mucositis and peri‐implantitis taxonomic microbial biomarkers at the community level, it was clear that mucositis is not only clinically but also from the taxonomic perspective an intermediate condition between health and peri‐implantitis. Functionally, albeit still significantly different in PERMANOVA, mucositis is microbially closer to peri‐implantitis, which is also evident from the slightly lower AUC for telling apart mucositis and peri‐implantitis using functional profiles as compared to taxonomic compositions (AUC = 0.78 vs. 0.84). This result, together with the lack of information in the literature on the mucositis‐associated species described here, points to a mucositis microbiome already having the metabolic capacity of contributing to disease. We can hypothesize that the transition towards the peri‐implantitis microbiome is at first (during mucositis) probably more driven by functional than taxonomic changes.

The highly consistent set of dominant submucosal species in peri‐implantitis samples allowed the identification of a strong microbial signature associated with this condition, reinforcing the notion of a major microbial role in the pathogenesis of peri‐implantitis. On the practical side, we leveraged this strong microbial signature to build highly accurate microbiome‐based machine‐learning models able to tell apart peri‐implant conditions. Moreover, the presence of two or more sites with bleeding and/or suppuration associated with mPPD ≥ 4 mm represents a risk condition with severe dysbiosis. Most cases of mucositis are characterized by these clinical parameters, highlighting the importance of early diagnosis and treatment of this disease condition to avoid its evolution into peri‐implantitis.

A limitation of the present study, inherent to the design, is the absence of prospective data to assess the evolution of mucositis cases and the stability of healthy cases. A second limitation is the recruitment of cases in a specific area of Italy, which may limit the generalizability of the present findings. Despite our study representing the current largest metagenomic investigation of the microbiome in peri‐implantitis, it will thus be crucial to further expand the study to larger sample sizes and more diverse host populations.

## 5 Conclusion

In conclusion, the associations presented precisely illustrate the importance of surveying the microbiome using high‐resolution techniques for finding meaningful associations with clinical conditions. Our work should then serve as the basis for further peri‐implant microbiome observational studies favouring shotgun metagenomic sequencing over 16S rRNA gene sequencing. This study should also encourage targeted investigations on the reported mucositis‐ and peri‐implantitis‐associated species. This will be key to validating our predictive models and fostering the development of novel preventive and therapeutic strategies for peri‐implant diseases.

## Author Contributions

P.G. and N.S. conceived and planned the study. P.G., C.T., N.S. and M.B. organized and supervised the sampling. P.G., C.T., A.B., F.D., E.D. and R.W. collected the samples. F.Ar. generated metagenomic data. D.B., V.H., F.As. and E.P. performed the bioinformatic analysis. D.B., V.H., E.P., N.S. and P.G. interpreted the analyses. P.G., V.H., E.P., D.B. and N.S. wrote the manuscript. C.T., L.T., M.B. and D.V. revised the text. All authors read and approved the final manuscript.

## Conflicts of Interest

P.G., M.B., N.S. and C.T. hold shares in PreBiomics S.r.l. E.P. and D.V. are consultants of PreBiomics S.r.l. D.B. and M.B. are employees of PreBiomics S.r.l.

## Supporting information


**Figure S1.** Comparison of beta‐diversity (intra‐group variability) between clinical groups based on taxonomic compositions (left panel) and functional profiles (right panel). Bray–Curtis dissimilarity index was used. Mann–Whitney *p*‐values: ** < 0.01; *** < 0.001.

**Figure S2.** Heatmap of the relative abundances (log scale) and the effect sizes (the LDA score from LEfSe) of the top 10 UniRef90 gene families with the highest effect sizes that characterize each study condition: health gene families (health vs. peri‐implantitis, white effect size bars), mucositis gene families (mucositis vs. health or peri‐implantitis, light‐grey effect size bars) and peri‐implantitis gene families (peri‐implantitis vs. health, dark‐grey effect size bars). The *p*‐values of the abundance test performed by LEfSe are reported inside the bar. The rectangular boxes represent the results of the prevalence test (Fisher exact test) between healthy and peri‐implantitis biomarkers.

**Figure S3.** Heatmap indicating the performance (in terms of mean AUC under the ROC curve values) of the random forest classifier in classifying samples into the study conditions based on clinical/demographic parameters alone, microbiome data alone (i.e., SGB relative abundances) and microbiome + clinical data combined.

**Table S1.** Characteristics of dental implants excluded during quality‐control (*n* = 11).
**Table S2.** Characteristics of the test dental implants analysed (*n* = 79).
**Table S3.** Characteristics of the contralateral dental implants/teeth analysed (*n* = 59).
**Table S4.** Metadata of implants analysed (*n* = 79).

## Data Availability

All metagenomes have been deposited and are available at the NCBI Sequence Read Archive under accession BioProject PRJNA547717.
