# Supplementary figures and images for: Shotgun Metagenomics Identifies in a Cross‐Sectional Setting Improved Plaque Microbiome Biomarkers for Peri‐Implant Diseases
Source: J Clin Periodontol. 2025 Jun 4;52(7):999–1010. doi: 10.1111/jcpe.14121 (PMC12176464; doi:10.1111/jcpe.14121)

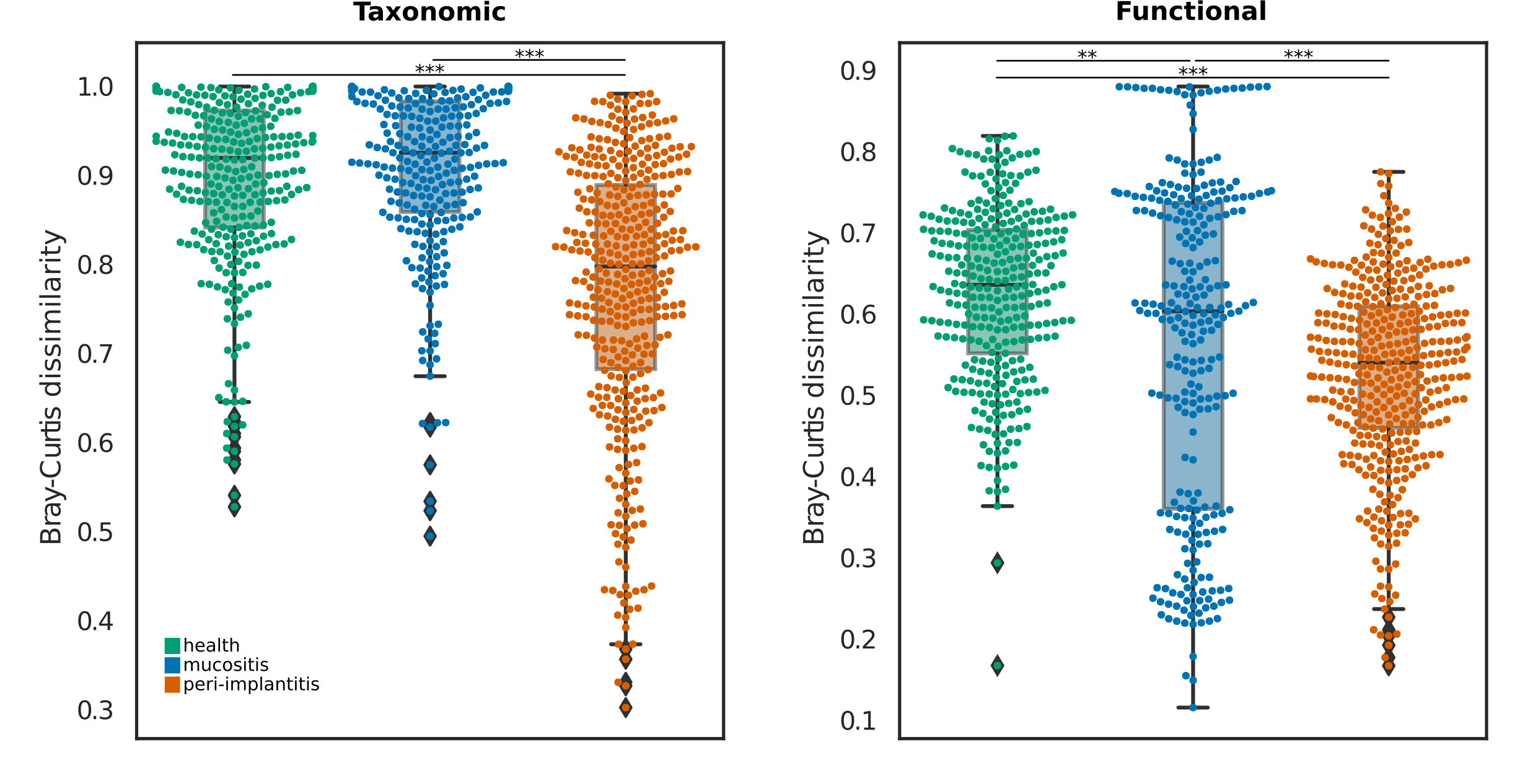

Supplement: Supplementary file 1 — Figure S1. Comparison of beta‐diversity (intra‐group variability) between clinical groups based on taxonomic compositions (left panel) and functional profiles (right panel). Bray–Curtis dissimilarity index was used. Mann–Whitney p‐values: ** < 0.01; *** < 0.001. [file JCPE-52-999-s004.png]

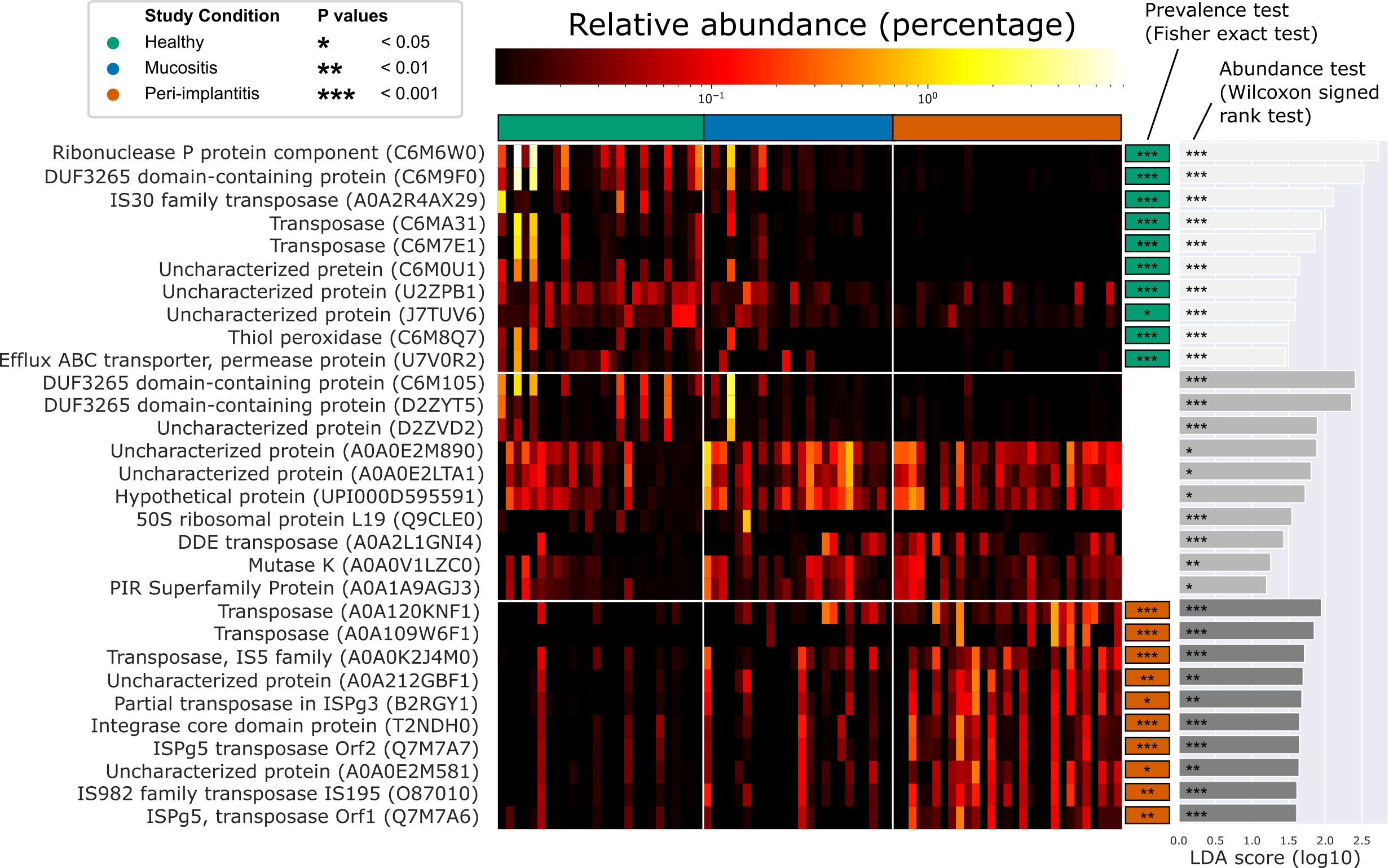

Supplement: Supplementary file 2 — Figure S2. Heatmap of the relative abundances (log scale) and the effect sizes (the LDA score from LEfSe) of the top 10 UniRef90 gene families with the highest effect sizes that characterize each study condition: health gene families (health vs. peri‐implantitis, white effect size bars), mucositis gene families (mucositis vs. health or peri‐implantitis, light‐grey effect size bars) and peri‐implantitis gene families (peri‐implantitis vs. health, dark‐grey effect size bars). The p‐values of the abundance test performed by LEfSe are reported inside the bar. The rectangular boxes represent the results of the prevalence test (Fisher exact test) between healthy and peri‐implantitis biomarkers. [file JCPE-52-999-s001.png]

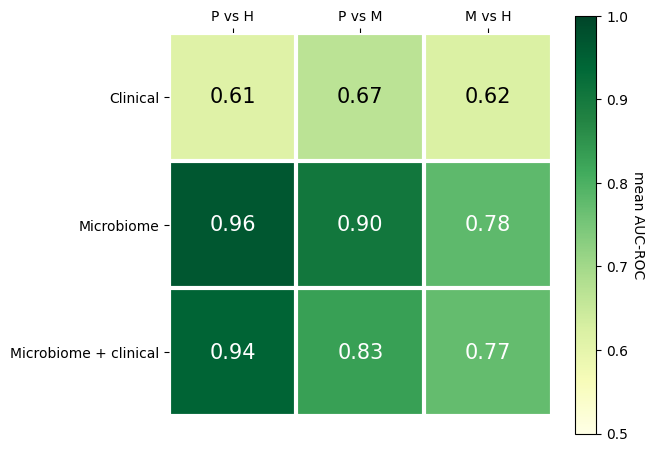

Supplement: Supplementary file 3 — Figure S3. Heatmap indicating the performance (in terms of mean AUC under the ROC curve values) of the random forest classifier in classifying samples into the study conditions based on clinical/demographic parameters alone, microbiome data alone (i.e., SGB relative abundances) and microbiome + clinical data combined. [file JCPE-52-999-s003.png]
